# Draft genome sequence of *Agarivorans albus* strain S3 capable of utilizing nori as a nutrient source

**DOI:** 10.1128/mra.00292-25

**Published:** 2025-05-22

**Authors:** Kiyohiko Seki, Yoshio Kawamura, Yukio Nagano

**Affiliations:** 1 Faculty of Agriculture, Saga University 13030, Saga, Japan; 2 Analytical Research Center for Experimental Sciences, Saga University 13030, Saga, Japan; California State University San Marcos, San Marcos, California, USA

**Keywords:** polysaccharide-degrading enzymes, marine bacteria

## Abstract

We sequenced the draft genome of *Agarivorans albus* strain S3, a bacterium capable of utilizing nori (*Pyropia yezoensis*), a polysaccharide-rich seaweed, as a nutrient source. The genome revealed approximately 100 genes involved in the degradation of starch, β-glucans including cellulose, galactans including agar/porphyran, mannans, xylan, and other polysaccharides.

## ANNOUNCEMENT

Nori (*Pyropia yezoensis*), an edible seaweed, contains a wide variety of polysaccharides, including porphyran in the intercellular spaces, xylan and mannan in the cell walls, and floridean starch as an energy reserve (1). Bacteria that can use this seaweed as a nutrient source may possess enzymes to degrade these complex polysaccharides. To explore this, we aimed to isolate such bacteria from mud samples collected near a seaweed cultivation site (33.196 N, 130.208 E). One gram of mud was suspended in 10 mL of artificial seawater with 1 g of dried seaweed powder and incubated statically at 20°C for 3 days. Following this, 1 mL of the suspension was transferred to fresh artificial seawater with the same seaweed concentration, and the process was repeated. The final suspension was plated on artificial seawater agar containing 1% dried seaweed, from which well-growing colonies were isolated. One colony, designated strain S3, was selected for draft genome sequencing.

The strain was cultured in a liquid medium containing 0.5% bacto tryptone, 0.1% yeast extract, 3% NaCl, 0.05% MgSO_4_·7H_2_O, 0.2% K_2_HPO_4_, and 0.04% KH_2_PO_4_, adjusted to pH 7.0. DNA extraction was performed using the NucleoSpin Microbial DNA kit (Macherey-Nagel, Germany). Sequencing libraries were prepared with the MGIEasy FS DNA Library Prep Set (MGI Tech, China), and 150 bp paired-end reads were obtained using a DNBSEQ-G400RS (MGI Tech) by Genome-Lead (Kagawa, Japan). Adapter sequences and low-quality bases were trimmed using fastp v0.23.4 with default parameters (2). Genome assembly was conducted with SPAdes v4.0.0, employing the --isolate parameter, and sequences under 200 bp were excluded (3). Gene annotation and taxonomic classification were performed using DFAST v1.6.0 with the “Perform Taxonomy Check” option to calculate average nucleotide identity (4). The quality of the genome assembly was evaluated using CheckM2 (5). Genes involved in polysaccharide degradation were identified using InterProScan v5.70 (6), with additional substrate predictions obtained from BLAST search results where needed.

The genome assembly results are presented in Table 1. Taxonomic analysis with DFAST confirmed that the strain belongs to *Agarivorans albus*. InterProScan, supplemented with BLAST where needed, identified a range of genes encoding polysaccharide-degrading enzymes: 24 genes for starch degradation, 18 genes for β-glucan degradation (including cellulose), 18 genes for galactan degradation (11 targeting agar/porphyran and 7 targeting other substrates), 10 genes for mannan degradation, 8 genes for xylan degradation, 1 gene likely encoding an exo-type chitinase, 9 genes encoding lysozymes, and 28 glycosyl hydrolase genes with unknown substrates.

**TABLE 1:** Assembly metrics and annotated features of *Agarivorans albus* strain S3

No. of filtered reads	Total sequence length (bp)	No. of contigs	Avg coverage (×)	N50 (bp)	No. of CDSs^*a*^	G + C content (%)	Completeness (%)	Contamination (%)
22,505,938	5,551,580	2,610	1,212	350,543	4,502	44.7	99.97	0.39

^*a*^ CDSs, coding DNA sequences.

In conclusion, *Agarivorans albus* strain S3, grown on nori as a nutrient source, possesses a diverse array of polysaccharide-degrading enzymes.

## Data Availability

The whole-genome sequencing project of *Agarivorans albus* strain S3 has been deposited at DDBJ/ENA/GenBank under the accession numbers GCA_047160015.1, BioProject accession number PRJDB19143, and BioSample accession number SAMD00829954. The raw sequences have been deposited in the SRA under the accession number DRR614324.
